# Coxsackievirus A21, Enterovirus 68, and Acute Respiratory Tract Infection, China

**DOI:** 10.3201/eid1805.111376

**Published:** 2012-05

**Authors:** Zichun Xiang, Richard Gonzalez, Zhong Wang, Lili Ren, Yan Xiao, Jianguo Li, Yongjun Li, Guy Vernet, Gláucia Paranhos-Baccalà, Qi Jin, Jianwei Wang

**Affiliations:** MOH Key Laboratory of Systems Biology of Pathogens, Beijing, People’s Republic of China (Z. Xiang, L. Ren, J. Li, Q. Jin, J. Wang);; Institute of Pathogen Biology, Beijing (Z. Xiang, R. Gonzalez, Z. Wang, L. Ren, Y. Xiao, J. Li, Q. Jin, J. Wang);; Fondation Mérieux, Lyon, France (R. Gonzalez, Y. Li, G. Vernet, G. Paranhos-Baccalà);; Peking Union Medical College Hospital, Beijing (Z. Wang)

**Keywords:** Coxsackievirus A21, enterovirus 68, adults, respiratory tract infection, enteroviruses, viruses, China

## Abstract

During August 2006–April 2010, in Beijing, China, 2 rare human enterovirus serotypes, coxsackievirus A21 and enterovirus 68, were detected most frequently in human enterovirus–positive adults with acute respiratory tract infections. Thus, during some years, these 2 viruses cause a substantial proportion of enterovirus-associated adult acute respiratory tract infections.

Human enteroviruses (HEVs) are small, nonenveloped RNA viruses belonging to the family *Picornaviridae*. HEVs are classified into 4 species (HEV-A to -D) according to their molecular and antigenic properties (*1*). HEVs are associated with diverse clinical syndromes, ranging from mild upper respiratory tract illnesses to severe and potentially fatal conditions, such as aseptic meningitis, encephalitis, myocarditis, acute flaccid paralysis, and hand-foot-and-mouth disease (*1*).

Although HEV serotypes can cocirculate, spatial and temporal factors determine the predominant serotype. For instance, in France and Spain, echovirus 11 and echovirus 6 are the predominant serotypes in patients with enterovirus respiratory infections (*2**,**3*). Clusters of the rare serotype enterovirus 68 (EV68), which causes severe respiratory infections in children, have been recently reported in the Philippines (*4*) and Japan (*5*). To help clinicians and public health officials better understand the epidemiologic and clinical profiles of HEV respiratory infections, temporal and geographic patterns of circulation, especially the dynamics of HEV serotype shift, need to be determined. We report that in some years in Beijing, People’s Republic of China, the rarely reported coxsackievirus A21 (CVA21) and EV68 are the predominant serotypes in adults with enterovirus-associated acute respiratory tract infection (ARTI).

## The Study

From August 2006 through April 2010, throat and nasal swabs were collected from 6,942 (3,158 male and 3,784 female) adult patients (>15 years of age) at the time of admission to the Fever Clinic Department at the Peking Union Medical College Hospital for ARTI. The 6,942 participants were randomly selected by physicians, as described (*6*). To detect HEV, we amplified 350–400 bp of the viral protein 1 (VP1) gene by reverse transcription PCR (*7*) and verified the findings by sequence analysis (GenBank accession nos. JN168998–JN169120). According to results of BLAST analysis (www.ncbi.nlm.nih.gov), the HEV detected in a sample was assigned to the serotype with which the amplified sequence shared >75% nt or >88% aa sequence identity (*8*). All samples were simultaneously screened for influenza viruses (A, B, and C), human parainfluenza viruses (1–4), respiratory syncytial virus, human coronaviruses (229E, NL63, HKU1, and OC43), metapneumovirus, adenovirus, and rhinovirus (*6*).

**Figure 1:**
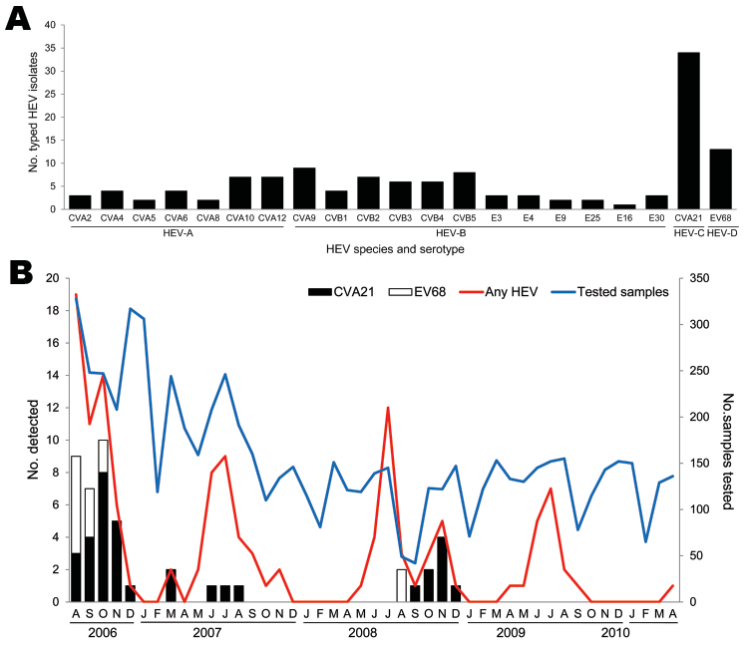
Frequency of human enterovirus (HEV) serotypes detected among adult patients by using sequence analysis of a partial viral protein 1 gene, in Beijing, People’s Republic of China, August 2006–April 2010. A) Number of patients detected for each HEV serotype; B) Seasonal distribution of the HEVs in adults with acute respiratory tract infection. Numbers of samples tested in each month during the study period are shown on the right-side y-axis. CV, coxsackievirus; E, echovirus; EV, enterovirus.

HEV was detected in 130 (69 male and 61 female) patients 15–70 years of age (median 27.5 years, mean 29.6 years). We identified 21 HEV serotypes. The most frequently detected serotypes were 2 that are rarely reported: CVA21 and EV68. The only HEV-C serotype detected in this study, CVA21, was found in 34 (26.2%) patients. The only HEV-D serotype detected in this study, EV68, was found in 13 (10%) patients. We also found 7 HEV-A serotypes and 12 HEV-B serotypes (Figure 1, panel A).

We detected 21 cases of CVA21 during August–December 2006 and 8 cases during September–December 2008, which accounted for 41.2% and 26.7% of all cases of HEVs, respectively. A few CVA21-positive cases were also detected in March and during June–August 2007. EV68 was detected in only 11 samples collected during August–October 2006 and in 2 samples in August 2008 (Figure 1, panel B; Table 1). The peaks of CVA21 and EV68 overlapped during August–October 2006. We co-detected other respiratory viruses in only 1 CVA21-positive patient and only 1 EV68-positive patient, indicating that the ARTIs in these patients are primarily associated with CVA21 or EV68 (Table 2).

**Table 1:** Yearly distribution of human enterovirus infections in adults with acute respiratory tract infection, People’s Republic of China, August 2006–April 2010

Date	Coxsackievirus A21, no. (%)	Enterovirus 68, no. (%)	Any human enterovirus, no.
2006 Aug–Dec	21 (41.2)	11 (21.6)	51
2007 Jan–Dec	5 (16.1)	0	31
2008 Jan–Dec	8 (26.7)	2 (6.7)	30
2009 Jan–Dec	0	0	17
2010 Jan–Apr	0	0	1
Total	34 (26.2)	13 (10.0)	130

**Table 2:** Demographic and clinical characteristics of sampled population with human enterovirus infections, People’s Republic of China, August 2006–April 2010*

Characteristic	No. (%) persons		
	Coxsackievirus A21, n = 34	Enterovirus 68, n = 13	Any human enterovirus, n = 130
Sex			
M	18 (52.9)	7 (53.8)	69 (53.1)
F	16 (47.1)	6 (46.2)	61 (46.9)
Signs and symptoms			
Pharyngeal congestion	34 (100)	13 (100.0)	128 (98.5)
Headache	27 (79.4)	10 (76.9)	104 (80.0)
Myalgia	25 (73.5)	9 (69.2)	93 (71.5)
Chills	25 (73.5)	7 (53.8)	89 (68.5)
Sore throat	23 (67.6)	7 (53.8)	82 (63.1)
Rhinorrhea	17 (50.0)	5 (38.5)	50 (38.5)
Sneezing	14 (41.2)	3 (23.1)	44 (33.8)
Cough	7 (20.6)	3 (23.1)	19 (14.6)
Swelling of tonsils	6 (17.6)	1 (7.7)	16 (12.3)
Expectoration	4 (11.8)	0	6 (4.6)
Rigors	1 (2.9)	1 (7.7)	4 (3.1)
Preliminary clinical diagnosis			
Upper respiratory tract infection	33 (97.1)	13 (100.0)	126 (96.9)
Tonsillitis	0	0	2 (1.5)
Pulmonary infection	0	0	1 (0.8)
Other viruses co-detected	1 (2.9)†	1 (7.7)†	8 (6.2)‡

*Median age (range) of patients with coxsackievirus A21 infection, 22 (15–67) y; median age (range) of patients with enterovirus V68 infection, 34 (18–67) y; median age (range) of patients with any enterovirus infection, 27.5 (15–70) y. Median age of patients with coxsackievirus A21 infection was younger than that of patients with other human enterovirus infections (χ^2^ 14.7; p<0.01). †Influenza virus A (n = 1). ‡Influenza virus A (n = 2), parainfluenza virus 3 (n = 2), rhinovirus (n = 2), metapneumovirus (n = 1), coronavirus OC43 (n = 1).

Major symptoms associated with HEV infection included pharyngeal congestion, headache, myalgia, chills, and sore throat but not increased respiratory rate or difficulty breathing. According to the Guidelines for Surveillance on Severe Acute Respiratory Infections at Sentinel Hospitals (2011) issued by the Chinese Ministry of Health (www.moh.gov.cn/publicfiles/business/htmlfiles/mohjbyfkzj/s3577/201102/50590.htm), ARTI patients >5 years of age who had increased respiratory rate (>25 breaths/min) or difficulty breathing were considered to have a severe ARTI. Our data showed that all CVA21- or EV68-positive patients identified in this study had mild ARTIs.

**Figure 2:**
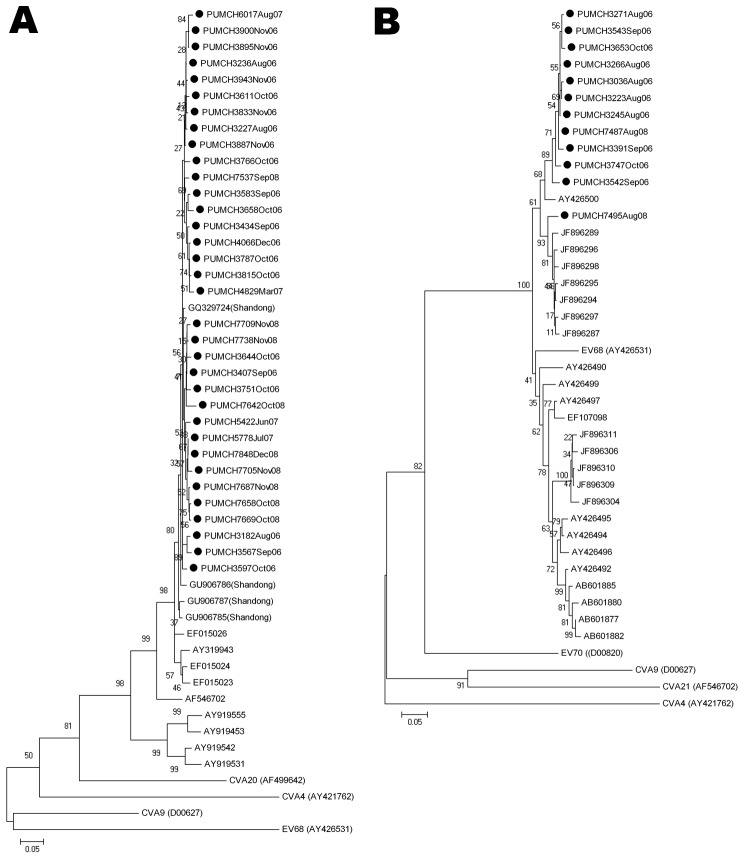
Phylogenetic analysis of human enteroviruses according to partial viral protein 1 (VP1) nucleotide sequences. The tree was generated with 1,000 bootstrap replicates. Neighbor-joining analysis of the targeted VP1 nucleotide sequence was performed by using the Kimura 2-parameter model with MEGA software version 4.0 (www.megasoftware.net). The scale bars indicate evolutionary distance. GenBank accession numbers for reference serotypes are indicated in parentheses. Each detected strain is indicated by black circles and a specific identification code followed by the patient number and the time of sampling. Strains detected by other research groups are indicated by a GenBank accession number. A) Phylogenetic tree of partial coxsackievirus (CV) A21 VP1 gene. The 375-bp fragments, which correspond to the locations of nt 2565–2939 of the CVA21 prototype strain (GenBank accession no. AF546702), were used to construct the tree. Enterovirus 68 (EV68), CVA9, CVA4, and CVA20 (GenBank accession nos. AY426531, D00627, AY421762, and AF499642, respectively) were used as outgroups. B) Phylogenetic tree of partial EV68 VP1 gene. The 390-bp fragments, which correspond to the locations of nt 2494–2883 of the EV68 prototype strain (GenBank accession no. AY426531), were used to construct the phylogenetic tree. CVA9, CVA4, CVA21, and EV70 (GenBank accession nos. D00627, AY421762, AF546702, and D00820, respectively) were used as outgroups.

The VP1 nucleotide sequences of the CVA21 and EV68 strains identified showed high sequence identity and were located in the same cluster in their phylogenetic trees, HEV-C and HEV-D, respectively (Figure 2). Nucleotide identity among the CVA21 strains identified was 95.6%–100% and that among EV68 strains was 92.3%–99.7%. However, the CVA21 and EV68 strains had low nucleotide identity compared with their prototype strains (CVA21, GenBank accession no. AF546702, 87%–89.2%; EV68, GenBank accession no. AY426531, 76.6%–78.9%). Alignment of the putative VP1 amino acid sequences of HEV strains with their respective prototype strains revealed a limited divergence; only 2 substitutions were found in CVA21, and 9 substitutions and 1 deletion was found in EV68 (data not shown). These findings suggest that the CVA21 or EV68 strains were similar but are changing and diverging from their prototype strains.

## Conclusions

The frequency of detecting CVA21 and EV68 among HEV-positive adults with ARTIs in Beijing, China, was high. The difference in the HEV serotypes we detected compared with serotypes detected by others (*2**,**3*) suggests that the spectrum of HEV serotypes associated with ARTIs differs among geographic regions.

Since isolation of these viruses, infections with CVA21 and EV68 have been rarely reported (*9**,**10*). In the United States, only 42 detections of CVA21 and 26 detections of EV68 have been reported during 36 years of HEV surveillance (*11*). However, EV68 was recently associated with ARTIs in France, the Netherlands, the United States, the Philippines, and Japan (*4**,**5**,**12**–**15*), indicating epidemics of EV68 are increasing in number and severity.

The reason for the high frequency of CVA21 and EV68 infections in Beijing is unclear. The EV68 strains identified in this study are probably novel strains or genetic variants to which the population is immune. Moreover, the deduced amino acid identity of the EV68 strains with strains detected in children in Japan (GenBank accession nos. AB601872–AB601885) (*5*) was 89.4%–93.8%, indicating the possible co-existence of 2 distinct phylogenetic lineages of EV68 strains (*13*). The emergence of new genetic lineages might be the reason for increasing activity of EV68 (*5**,**13*). Because the median age of CVA21-positive patients was significantly lower than that of other HEV-positive patients (Table 2), we suspect that years of CVA21quiescence probably resulted in increased susceptibility of the younger population (*11*). Outbreaks of classical HEVs tend to occur in several-year cycles (*11*). We noted that CVA21 infections were concentrated in 2006 and 2008. However, because patients were not enrolled in our study after April 2010, our data on HEV circulation might be incomplete. Longer surveillance would be useful for determining the cycle of CVA21 and EV68 infections. Another limitation of this study is that samples were collected from outpatients but not hospitalized patients, which could bias the classification of illness toward being mild. However, this study presents evidence that CVA21 and EV68 cause mild disease and fills a void left by other studies that included only hospitalized patients.

In conclusion, we provide evidence that during some years in adults in Beijing, China, the rarely reported HEV serotypes CVA21 and EV68 are responsible for a substantial proportion of enterovirus-associated ARTIs. Our findings provide further insight into the pathogenesis of HEVs and increase our awareness of their clinical role. To predict possible outbreaks, global surveillance and investigation of HEV serotypes in patients with ARTIs should be strengthened.

## References

[1] Pallansch M, Roos R. Enteroviruses: polioviruses, coxsackieviruses, echoviruses, and newer enteroviruses. In: Knipe DM, Howley PM, editors. Fields virology, 5th ed. Philadelphia: Lippincott Williams & Wilkins; 2007.

[2] Jacques J, Moret H, Minette D, Lévêque N, Jovenin N, Deslée G, Epidemiological, molecular, and clinical features of enterovirus respiratory infections in French children between 1999 and 2005. J Clin Microbiol. 2008;46:206–13. 10.1128/JCM.01414-07 18003804 PMC2224256

[3] Trallero G, Avellon A, Otero A, De Miguel T, Pérez C, Rabella N, Enteroviruses in Spain over the decade 1998–2007: virological and epidemiological studies. J Clin Virol. 2010;47:170–6. 10.1016/j.jcv.2009.11.013 20007023

[4] Imamura T, Fuji N, Suzuki A, Tamaki R, Saito M, Aniceto R, Enterovirus 68 among children with severe acute respiratory infection, the Philippines. Emerg Infect Dis. 2011;17:1430–5. 21801620 10.3201/eid1708.101328 PMC3381551

[5] Kaida A, Kubo H, Sekiguchi J, Kohdera U, Togawa M, Shiomi M, Enterovirus 68 in children with acute respiratory tract infections, Osaka, Japan. Emerg Infect Dis. 2011;17:1494–7. 21801632 10.3201/eid1708.110028 PMC3381549

[6] Ren L, Gonzalez R, Wang Z, Xiang Z, Wang Y, Zhou H, Prevalence of human respiratory viruses in adults with acute respiratory tract infections in Beijing, 2005–2007. Clin Microbiol Infect. 2009;15:1146–53. 10.1111/j.1469-0691.2009.02746.x 19456830 PMC7129754

[7] Nix WA, Oberste MS, Pallansch MA. Sensitive, seminested PCR amplification of VP1 sequences for direct identification of all enterovirus serotypes from original clinical specimens. J Clin Microbiol. 2006;44:2698–704. 10.1128/JCM.00542-06 16891480 PMC1594621

[8] Oberste MS, Maher K, Kilpatrick DR, Pallansch MA. Molecular evolution of the human enteroviruses: correlation of serotype with VP1 sequence and application to picornavirus classification. J Virol. 1999;73:1941–8. 9971773 10.1128/jvi.73.3.1941-1948.1999 PMC104435

[9] Lennette EH, Fox VL, Schmidt NJ, Culver JO. The Coe virus: an apparently new virus recovered from patients with mild respiratory disease. Am J Hyg. 1958;68:272–87. 13594929

[10] Schieble JH, Fox VL, Lennette EH. A probable new human picornavirus associated with respiratory diseases. Am J Epidemiol. 1967;85:297–310. 4960233 10.1093/oxfordjournals.aje.a120693

[11] Khetsuriani N, Lamonte–Fowlkes A, Oberst S, Pallansch MA. Enterovirus surveillance—United States, 1970–2005. MMWR Surveill Summ. 2006;55:1–20. 16971890

[12] Petitjean–Lecherbonnier J, Dina J, Nguyen E, Gouarin S, Lebigot E, Vabret A. Molecular diagnosis of respiratory enterovirus infections: use of PCR and molecular identification for a best approach of the main circulating strains during 2008. Pathol Biol (Paris). 2011;59:113–21. 20828940 10.1016/j.patbio.2010.07.010 PMC7126958

[13] Rahamat–Langendoen J, Riezebos–Brilman A, Borger R, van der Heide R, Brandenburg A, Schölvinck E, Upsurge of human enterovirus 68 infections in patients with severe respiratory tract infections. J Clin Virol. 2011;52:103–6. 10.1016/j.jcv.2011.06.019 21802981

[14] Centers for Disease Control and Prevention. Clusters of acute respiratory illness associated with human enterovirus 68—Asia, Europe, and United States, 2008–2010. MMWR Morb Mortal Wkly Rep. 2011;60:1301–4. 21956405

[15] Hasegawa S, Hirano R, Okamoto-Nakagawa R, Ichiyama T, Shirabe K. Enterovirus 68 infection in children with asthma attacks: virus-induced asthma in Japanese children. Allergy. 2011;66:1618–20. 10.1111/j.1398-9995.2011.02725.x 21958204

